# Impact of Baseline Atrial Fibrillation on Conduction Disturbances After TAVR: Insights from a Large Cohort Study

**DOI:** 10.3390/jcm14217705

**Published:** 2025-10-30

**Authors:** Ziad Arow, Omar Oliva, Laurent Bonfils, Laurent Lepage, Hana Vaknin-Assa, Abid Assali, Didier Tchetche, Nicolas Dumonteil

**Affiliations:** 1 Groupe CardioVasculaire Interventionnel, Clinique Pasteur, 31300 Toulouse, France; ziad.arow@gmail.com (Z.A.); omaroliva93@gmail.com (O.O.); lbonfils@clinique-pasteur.com (L.B.); laulepage@yahoo.fr (L.L.); dtchetche@clinique-pasteur.com (D.T.); 2 Cardiology Department, Meir Medical Center, Tel Aviv University, Kfar Saba 44281, Israel; hana100niki@gmail.com (H.V.-A.); aassali@clalit.org.il (A.A.)

**Keywords:** transcatheter aortic valve replacement (TAVR), atrial fibrillation (AF), sinus rhythm (SR), permanent pacemaker (PPM) implantation, left bundle branch block (LBBB)

## Abstract

**Background**: Pre-existing atrial fibrillation (AF) is common among patients undergoing transcatheter aortic valve replacement (TAVR). However, evidence regarding its impact on the risk of permanent pacemaker (PPM) implantation and other conduction disturbances (CDs) after TAVR remains inconsistent. The aim of this study was to assess the effect of baseline heart rhythm on the risk of conduction abnormalities following TAVR. **Methods**: This study included patients with severe AS who underwent TAVR using either balloon-expandable (BEVs) or self-expanding valves (SEVs). The primary endpoint was the incidence of PPM implantation and new or worsening left bundle branch block (LBBB) after TAVR according to baseline rhythm (sinus rhythm vs. AF). Secondary endpoints were predictors of PPM implantation, LBBB, the occurrence of periprocedural stroke, and in-hospital mortality. **Results**: A total of 5195 TAVR patients were included: 3560 with baseline sinus rhythm and 1635 with baseline AF. PPM implantation was more frequent in patients with AF than in those with sinus rhythm (17% vs. 15%, *p* = 0.033), whereas new or worsening LBBB was less common (11% vs. 14%, *p* = 0.026). After adjustment with multivariable logistic regression, these associations were no longer statistically significant (PPM implantation: OR 1.156, 95% CI 0.969–1.379, *p* = 0.108; new or worsening LBBB: OR 0.826, 95% CI 0.676–1.010, *p* = 0.062). Independent peri-procedural predictors of PPM implantation included baseline first-degree AV block, pre-procedural RBBB, the use of self-expandable valves, implantation of larger valve sizes (≥23 mm), and the need for valve repositioning. **Conclusions**: In this large cohort, baseline AF was not associated with an increased risk of PPM implantation or new onset LBBB compared with sinus rhythm. These findings suggest that baseline rhythm alone should not be considered an independent predictor of PPM implantation or CDs following TAVR.

## 1. Introduction

Transcatheter aortic valve replacement (TAVR) has become a life-saving intervention for patients with severe aortic stenosis (AS) [1,2,3]. The number of TAVR procedures has increased substantially over the past decade and continues to expand worldwide as indications broaden and technology advances [4,5]. Atrial fibrillation (AF) is one of the most common cardiac arrhythmias, with a lifetime risk estimated at nearly one in three individuals. The prevalence of AF is expected to double in the next few decades as a result of the ageing population, increasing burden of comorbidities, and new technologies for detection [6,7]. A considerable proportion of patients undergoing TAVR present with pre-existing AF, reflecting the overlap between advanced age, structural heart disease, and atrial arrhythmogenesis [8]. Conduction disturbances (CDs), particularly high-degree atrioventricular block requiring permanent pacemaker (PPM) implantation, remain among the most frequent complications of TAVR [9]. While there are several clinical and procedural predictors of PPM after TAVR have been established, such as baseline right bundle branch block, valve type, implantation depth, and valve oversizing [9,10,11,12], the role of baseline cardiac rhythm is less well defined. Specifically, the impact of AF compared with sinus rhythm on post-TAVR conduction outcomes has been inconsistently reported across studies [13,14,15,16], with some suggesting slightly higher risk and others showing no independent association. Moreover, only a limited number of studies have specifically evaluated the effect of baseline AF versus sinus rhythm on the risk of conduction abnormalities and PPM implantation. The present study aims to clarify the influence of baseline AF on the incidence of new CDs and PPM implantation following TAVR in a large contemporary cohort.

## 2. Methods

This retrospective, single-center clinical registry was conducted at Clinique Pasteur (Toulouse, France). Data were collected from all patients undergoing TAVR for severe aortic stenosis using balloon (Edwards Sapien 3, Sapien 3 Ultra, Colibri, and MyVal) or self-expandable (Medtronic Evolut R, Evolut Pro, Evolut Pro+, Navitor, and ACURATE neo) valves (SEVs) between 2013 and 2024. Patient selection for TAVI followed the European Society of Cardiology guidelines for valvular heart disease [1] and was determined by a multidisciplinary Heart Team. All consecutive patients with documented baseline rhythm were included, while those with a pre-existing PPM, without available ECG data, or undergoing TAVR for isolated aortic regurgitation were excluded. Missing data were minimal and handled using complete-case analysis. Information on AF subtype (paroxysmal, persistent, or permanent) was not available, and all AF patients were analyzed as a single group. All patients provided informed consent for the use of their medical records for research purposes.

The primary endpoint of this study was the incidence of PPM implantation and new or worsening left bundle branch block (LBBB) after TAVR according to baseline rhythm (sinus rhythm vs. AF). New LBBB was defined as a post-procedural QRS duration ≥ 120 ms with typical LBBB morphology that was not present before the procedure, while worsening LBBB was defined as pre-existing LBBB with a post-procedural QRS duration increase to >150 ms. Decisions regarding permanent pacemaker implantation followed the European Society of Cardiology (ESC) Guidelines on Cardiac Pacing and Cardiac Resynchronization Therapy [17], ensuring standardized indications across operators. Secondary endpoints included identification of predictors of PPM implantation and new or worsening LBBB, as well as the occurrence of periprocedural stroke, in-hospital mortality and bleeding complications according to baseline rhythm following TAVR.

## 3. Statistical Analysis

Categorical and dichotomous variables are presented as frequencies and percentages and were compared using Pearson’s chi-square or Fisher’s exact tests, as appropriate.

The Kruskal–Wallis test was used to assess the distribution of continuous variables. Continuous variables with a normal distribution are reported as mean ± standard deviation and were compared using the unpaired, two-sided Student’s *t*-test. Non-normally distributed variables are reported as median and interquartile range and were compared using the Mann–Whitney U test.

Univariate and multivariate logistic regression analyses were performed to identify factors associated with PPM implantation and new or worsening LBBB, using a backward stepwise method including variables with *p* < 0.20 in univariate analysis. Potential collinearity between AF and baseline conduction parameters (first degree AV block, RBBB and LBBB) was assessed using variance inflation factors (VIFs); all VIF values were <5, indicating no significant multicollinearity. All *p*-values were two-sided, and values < 0.05 were considered statistically significant. Analyses were conducted using SPSS version 29.0.2.0 (IBM Corp., Armonk, NY, USA).

## 4. Results

A total of 5195 patients who underwent TAVR were included, comprising 3560 with baseline sinus rhythm and 1635 with baseline AF. Baseline characteristics are summarized in Table 1. The median age was 84 years in both groups. The AF group included a higher proportion of males compared with the sinus rhythm group (56% vs. 50%). Cardiovascular comorbidities were prevalent in both groups, including dyslipidemia, hypertension, diabetes mellitus, and coronary artery disease. In the sinus rhythm group, 13% of patients had first-degree AV block. The prevalence of preprocedural RBBB was similar between groups (13% vs. 14%), whereas patients with AF had a higher prevalence of preprocedural LBBB (14% vs. 10%). The mean aortic gradient was slightly higher in the sinus rhythm group (48 mmHg vs. 44 mmHg), whereas patients with baseline AF had a higher prevalence of mitral regurgitation (54% vs. 43%, *p* < 0.001). Both the Society of Thoracic Surgeons (STS) score and EuroSCORE were higher in the AF group (median 4.1 and 3.9, respectively) compared with the sinus rhythm group (3.5 and 3, respectively).

Table 2 summarizes the Peri-procedural data and in hospital outcomes. The use of balloon and self-expandable valves was similar between groups. Larger valve sizes (≥23 mm) were more frequently implanted in the AF group (92% vs. 87%, *p* < 0.001). Procedure duration, fluoroscopy time, and contrast volume were comparable between groups. Pre-dilatation was performed less often in AF patients (14% vs. 18%, *p* = 0.009), whereas valve repositioning was more frequent (25% vs. 27%, *p* = 0.015). Rates of post-dilatation (23% vs. 22%, *p* = 0.377) and the need for a second valve (1.1% vs. 0.95%, *p* = 0.632) did not differ significantly between groups.

**Figure 1:**
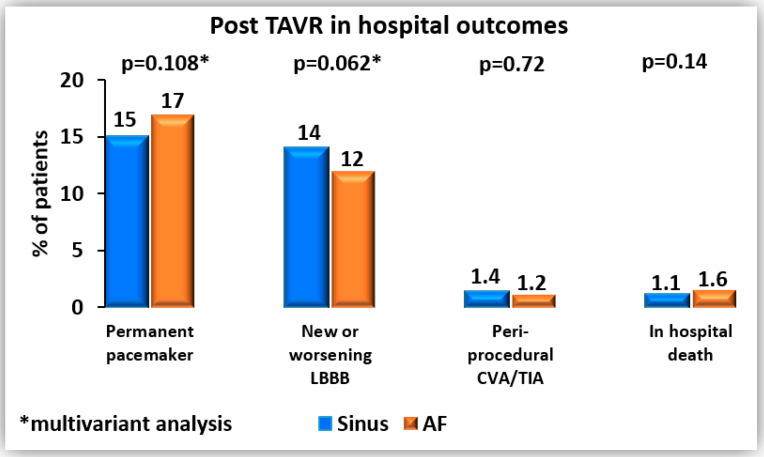
Post-TAVR in hospital outcomes.

In terms of in-hospital outcomes (Figure 1), permanent pacemaker implantation was more frequent in patients with AF compared with sinus rhythm (17% vs. 15%, *p* = 0.033), while new or worsening LBBB was less common (11% vs. 14%, *p* = 0.026). However, after multivariable logistic regression analysis (Table 3a,b), these associations were no longer statistically significant. Independent peri-procedural predictors of permanent pacemaker implantation included baseline first-degree AV block, pre-procedural RBBB, the use of self-expandable valves compared with balloon-expandable valves, implantation of larger valve sizes (≥23 mm), and the need for valve repositioning. For new or worsening LBBB, the only independent peri-procedural predictor was implantation of a larger valve size (≥23 mm).

Rates of peri-procedural stroke were low and similar between groups (1.2% vs. 1.4%, *p* = 0.729). In-hospital mortality was also comparable, at 1.6% in the AF group and 1.1% in the sinus rhythm group (*p* = 0.142) (Table 2). No statistically significant difference was observed between the groups in life-threatening bleeding complications (1.2% in the sinus rhythm group vs. 1.6% in the AF group, *p* = 0.232) or major bleeding complications (3.2% vs. 3.3%, *p* = 0.763).

## 5. Discussion

In this large study, pre-procedural AF, compared with sinus rhythm, was not associated with an increased risk of permanent pacemaker implantation or new LBBB. Rates of peri-procedural stroke, in-hospital mortality and bleeding complications were low and similar between groups.

New-onset AF after TAVR is associated with worse clinical outcomes, including higher mortality and increased risk of permanent pacemaker implantation [18,19]. In contrast, the effect of baseline rhythm (AF versus sinus rhythm) on pacemaker risk following TAVR remains unclear and inconsistently reported in the literature. Mentias et al. [13] found that pre-existing AF was not independently associated with an increased risk of PPM implantation after TAVR and the study highlights that the role of baseline rhythm in post-TAVR CDs remains unclear and inconsistently reported. Patil et al. [14] evaluated the impact of AF on inpatient outcomes after TAVR and found that AF was not associated with PPM implantation. Khan et al. [15] reported that baseline AF and pre-existing LBBB were independent predictors of late PPM implantation following TAVR. A very large meta-analysis [16] found that pre-existing conduction abnormalities and AF are associated with increased risk of PPM after TAVR. Our study contributes further data suggesting that baseline AF is not associated with an increased incidence of permanent pacemaker implantation or new LBBB after TAVR.

The higher mortality reported in the literature among patients with baseline AF likely reflects the presence of atrial myopathy and global myocardial remodeling, which are markers of advanced cardiac disease and increased vulnerability to adverse outcomes [20,21]. However, these structural and electrophysiological changes do not directly involve the conduction system injured during TAVR, explaining why AF was not independently associated with new CDs.

These findings have important clinical implications, suggesting that AF alone should not be considered a high-risk feature for post-procedural CDs. Procedural planning and patient counseling should therefore focus on other well-established predictors, including baseline first-degree AV block, pre-procedural RBBB, the use of self-expandable valves, implantation of larger valve sizes (≥23 mm), and the need for valve repositioning, factors that were identified as independent predictors of pacemaker implantation in our study, rather than focusing on AF. Consistent with prior evidence, our data confirmed a higher rate of PPM implantation with self-expanding valves compared with balloon-expandable valves. This difference is well recognized in the literature and is primarily attributed to deeper valve positioning and sustained radial force exerted by self-expanding prostheses on the interventricular septum, which increase the risk of conduction system injury [10,22,23]. Moreover, unnecessary heightened monitoring or prophylactic measures based solely on AF may be avoided, optimizing hospital resources and shortening unnecessary prolonged hospital stays.

This study has several limitations that warrant consideration. First, because it is a retrospective observational analysis, there is a risk of confounding factors and bias influencing the results. In particular, potential confounding by unmeasured variables such as medication use (e.g., antiarrhythmics) and procedural factors including valve type or implantation depth cannot be excluded. Second, the study included different generations and types of transcatheter valves, which may have varying impacts on CDs. Third, even though our patient cohort is relatively large, the fact that this was conducted at a single center may limit the generalizability of the findings to other institutions with different patient populations and procedural techniques. Finally, we focused only on in-hospital outcomes and did not capture longer-term events such as late pacemaker implantation.

In conclusion, in this large cohort, baseline atrial fibrillation was not associated with an increased risk of permanent pacemaker implantation or new-onset LBBB, suggesting that baseline rhythm alone is not an independent predictor of post-TAVR conduction disturbances.

## Figures and Tables

**Table 1:** Baseline characteristics.

Characteristic	Sinus Rhythm	Atrial Fibrillation	*p*-Value
n	3560	1635	
Age, years, median (Q1–Q3)	84 (80–87)	84 (81–88)	<0.001
Gender, male n (%)	1789 (50)	912 (56)	<0.001
Cardiovascular Comorbidities			
Dyslipidemia, n (%)	1676 (47)	714 (44)	0.020
Hypertension, n (%)	2805 (78)	1265 (77)	0.386
Diabetes mellitus, n (%)	296 (22)	400 (24)	0.100
BMI (mean ± SD)	26 ± 4	26 ± 4	0.020
BSA (mean ± SD)	1.7 ± 0.2	1.7 ± 0.2	<0.001
Coronary artery disease, n (%)	1544 (43)	674 (41)	0.272
Prior PCI, n (%)	1069 (30)	483 (30)	0.708
Prior CABG, n (%)	181 (5)	103 (6)	0.075
Prior CVA/TIA, n (%)	248 (7)	191 (12)	<0.001
PVD, n (%)	397 (11)	217 (13)	0.029
Baseline ECG			
First degree AV block	476 (13)	N/A	N/A
RBBB	475 (13)	233 (14)	0.380
LBBB	368 (10)	230 (14)	<0.001
Baseline TTE and CT Data			
LVEF%, median (Q1–Q3)	63 (56–67)	60 (50–65)	<0.001
AVA, cm^2^ (mean ± SD)	0.9 ± 0.3	0.78 ± 0.2	0.588
AVA Index, (mean ± SD)	0.58 ± 0.2	0.44 ± 0.13	0.588
Mean Aortic gradient, mmHg, (mean ± SD)	48 ± 13	44 ± 13	<0.001
Aortic regurgitation (Any), n (%)	1088 (30)	540 (33)	0.078
Mitral regurgitation (Any), n (%)	1533 (43)	895 (54)	<0.001
Calcium score, AU (mean ± SD)	2869 ± 3452	2728 ± 3404	0.188
Surgical Risk			
Euroscore II, median (Q1-Q3)	3.02 (1.97–4.94)	3.96 (2.34–6.47)	<0.001
STS Score, median (Q1-Q3)	3.52 (2.33–5.30)	4.12 (2.72–6.29)	<0.001

BMI = Body Mass Index; PCI = Percutaneous coronary intervention; CABG = Coronary artery bypass graft; CVA = Cerebrovascular Accident; TIA = Transient ischemic attack; PVD = Peripheral vascular disease; LVEF = Left ventricle ejection fraction; AVA = Aortic valve area; AU = Agatston Unit.

**Table 2:** Peri-procedural data and in hospital outcomes.

	Sinus Rhythm	Atrial Fibrillation	*p*-Value
n	3560	1635	
BEVs, n (%)	1072 (30)	519 (32)	0.242
SEVs, n (%)	2488 (70)	1116 (68)	0.242
Valve size > 23 mm, n (%)	3094 (87)	1500 (92)	<0.001
Procedure duration, min (mean ± SD)	47 ± 18	47 ± 19	0.875
Fluoroscopy time, min (mean ± SD)	13 ± 8	12 ± 6	0.560
Contrast medium, ml (mean ± SD)	85 ± 37	83 ± 36	0.037
Pre-dilatation, n (%)	636 (18)	233 (14)	0.009
Post-dilatation, n (%)	777 (22)	375 (23)	0.377
Valve repositioning, n (%)	987 (27)	403 (25)	0.015
Need for second valve, n (%)	34 (0.95)	18 (1.1)	0.632
In hospital outcomes			
Permanent Pacemaker, n (%)	552 (15)	292 (17)	0.033
New or worsening LBBB, n (%)	485 (14)	187 (11)	0.026
Peri-procedural CVA, n (%)	50 (1.4)	21 (1.2)	0.729
In hospital death, n (%)	41 (1.1)	27 (1.6)	0.142
Life-threatening bleeding, n (%)	44 (1.2)	27 (1.6)	0.232
Major bleeding, n (%)	114 (3.2)	55 (3.3)	0.763

BEV = Balloon-expandable valves; SEVs = Self-expandable Valves; CVA = Cerebrovascular accident.

**Table 3:** (**a**) Permanent pacemaker. (**b**) New or worsening LBBB.

(a)				
	Univariable OR (95% CI)	*p*-Value	Multivariable OR (95% CI)	*p*-Value
Age,	1.017 (95% CI, 1.004–1.030)	0.011	1.016 (95% CI, 1.001–1.032)	0.041
Gender, male	1.273 (95% CI, 1.097–1.477)	0.001	1.131 (95% CI, 0.907–1.410)	0.275
Dyslipidemia	0.885 (95% CI, 0.763–1.027)	0.107	0.846 (95% CI, 0.715–1.002)	0.052
Hypertension	1.163 (95% CI, 0.967–1.399)	0.108	1.063 (95% CI, 0.865–1.306)	0.564
Diabetes mellitus	1.182 (95% CI, 0.997–1.402)	0.054	1.205 (95% CI, 0.993–1.462)	0.060
BMI	1.019 (95% CI, 1.004–1.034)	0.011	1.020 (95% CI, 0.997–1.044)	0.093
BSA	1.691 (95% CI, 1.199–2.384)	0.003	0.862 (95% CI, 0.451–1.649)	0.654
Coronary artery disease	0.990 (95% CI, 0.853–1.149)	0.898	-	-
Prior PCI	1.005 (95% CI, 0.855–1.180)	0.953	-	-
Prior CABG	0.941 (95% CI, 0.676–1.309)	0.719	-	-
Prior CVA/TIA	1.218 (95% CI, 0.950–1.561)	0.120	1.074 (95% CI, 0.787–1.392)	0.754
PVD	1.100 (95% CI, 0.880–1.375)	0.402	-	-
First degree AV block	1.855 (95% CI, 1.520–2.264)	<0.001	1.478 (95% CI, 1.174–1.861)	<0.001
RBBB	5.289 (95% CI, 4.445–6.294)	<0.001	4.744 (95% CI, 3.919–5.742)	<0.001
LBBB	0.900 (95% CI, 0.794–1.211)	0.125	-	-
LVEF%	1.003 (95% CI, 0.996–1.009)	0.408	-	-
AVA, cm^2^	1.625 (95% CI, 1.110–2.378)	0.012	1.481 (95% CI, 0.939–2.337)	0.092
Mean Aortic gradient, mmHg	0.995 (95% CI, 0.990–1.001)	0.105	0.997 (95% CI, 0.990–1.004)	0.364
Calcium score, AU	1.000 (95% CI, 1.000–1.000)	0.568	-	-
Euroscore II	1.000 (95% CI, 0.984–1.017)	0.964	-	-
STS Score	1.005 (95% CI, 0.984–1.026)	0.662	-	-
Atrial fibrillation at baseline	1.184 (95% CI 1.013–1.383)	0.034	1.156 (95% CI, 0.969–1.379)	0.108
Valve type (BEV vs. SEV)	0.795 (95% CI, 0.673–0.938)	0.006	0.812 (95% CI, 0.659–1.000)	0.050
Valve Size (>23 vs. ≤23)	1.752 (95% CI, 1.335–2.300)	<0.001	1.566 (95% CI, 1.140–2.153)	0.006
Pre-Dilation	0.981 (95% CI, 0.804–1.196)	0.847	-	-
Post-Dilation	1.118 (95% CI, 0.939–1.330)	0.209	-	-
Valve repositioning	1.219 (95% CI, 1.037–1.434)	0.017	1.276 (95% CI, 1.054–1.544)	0.012
Need for a second valve	1.229 (95% CI, 0.614–2.460)	0.560	-	
(**b**)				
	**Univariable OR (95% CI)**	***p*-Value**	**Multivariable OR (95% CI)**	***p*-Value**
Age,	0.986 (95% CI, 0.973–0.999)	0.033	0.984 (95% CI, 0.969–0.999)	0.040
Gender, male	0.909 (95% CI, 0.773–1.069)	0.249	-	
Dyslipidemia	0.991 (95% CI, 0.842–1.166)	0.915	-	
Hypertension	0.896 (95% CI, 0.739–1.087)	0.266	-	
Diabetes mellitus	1.096 (95% CI, 0.907–1.324)	0.344	-	
BMI	1.007 (95% CI, 0.991–1.024)	0.402	-	
BSA	1.025 (95% CI, 0.700–1.501)	0.897	-	
Coronary artery disease	0.897 (95% CI, 0.761–1.058)	0.197	1.009 (95% CI, 0.782–1.302)	0.944
Prior PCI	0.821 (95% CI, 0.684–0.986)	0.035	0.886 (95% CI, 0.672–1.168)	0.390
Prior CABG	1.080 (95% CI, 0.763–1.530)	0.664	-	
Prior CVA/TIA	0.759 (95% CI, 0.553–1.042)	0.089	0.758 (95% CI, 0.533–1.077)	0.122
PVD	0.792 (95% CI, 0.605–1.037)	0.090	0.813 (95% CI, 0.597–1.008)	0.189
First degree AV block	1.197 (95% CI, 0.943–1.518)	0.139	1.139 (95% CI, 0.872–1.488)	0.341
RBBB	-	-	-	-
LVEF%	1.008 (95% CI, 1.001–1.016)	0.031	1.006 (95% CI, 0.996–1.015)	0.239
AVA, cm^2^	1.340 (95% CI, 0.878–2.046)	0.175	1.487 (95% CI, 0.888–2.489)	0.063
Mean Aortic gradient, mmHg	1.004 (95% CI, 0.998–1.010)	0.177	1.005 (95% CI, 0.997–1.012)	0.243
Euroscore II	0.973 (95% CI, 0.952–0.994)	0.012	0.990 (95% CI, 0.962–1.019)	0.510
STS Score	0.974 (95% CI, 0.948–1.000)	0.053	1.010 (95% CI, 0.979–1.042)	0.524
Atrial fibrillation at baseline	0.813 (95% CI, 0.679–0.973)	0.024	0.826 (95% CI, 0.676–1.010)	0.062
Valve type (BEV vs. SEV)	0.781 (95% CI, 0.650–0.938)	0.008	0.816 (95% CI, 0.653–1.020)	0.074
Valve Size (>23 vs. ≤23)	1.445 (95% CI, 1.088–1.919)	0.011	1.448 (95% CI, 1.050–1.997)	0.024
Pre-Dilation	0.854 (95% CI, 0.681–1.071)	0.171	0.780 (95% CI, 0.612–1.002)	0.052
Post-Dilation	1.104 (95% CI, 0.912–1.337)	0.311	-	-
Valve repositioning	1.084 (95% CI, 0.905–1.299)	0.381	-	-
Need for a second valve	0.410 (95% CI, 0.127–1.319)	0.135	0.475 (95% CI, 0.146–1.543)	0.215

BMI = Body Mass Index; BSA = Body Surface Area; PCI = Percutaneous coronary intervention; CABG = Coronary artery bypass graft; CVA = Cerebrovascular Accident; TIA = Transient ischemic attack; PVD = Peripheral vascular disease; LVEF = Left ventricle ejection fraction; AVA = Aortic valve area; AU = Agatston Unit.

## Data Availability

The datasets used and/or analysed during the current study are available from the corresponding author on reasonable request.
